# Regression-Based Camera Pose Estimation through Multi-Level Local Features and Global Features

**DOI:** 10.3390/s23084063

**Published:** 2023-04-18

**Authors:** Meng Xu, Zhihuang Zhang, Yuanhao Gong, Stefan Poslad

**Affiliations:** 1 School of Electronic Engineering and Computer Science, Queen Mary University of London, London E1 4NS, UK; meng.xu@qmul.ac.uk; 2 School of Vehicle and Mobility, Tsinghua University, Beijing 100084, China; zhihuang18@mails.tsinghua.edu.cn; 3 College of Electronics and Information Engineering, Shenzhen University, Shenzhen 518061, China; gong@szu.edu.cn

**Keywords:** pose estimation, image matching, local feature, global feature, deformable network, geometric constraint

## Abstract

Accurate and robust camera pose estimation is essential for high-level applications such as augmented reality and autonomous driving. Despite the development of global feature-based camera pose regression methods and local feature-based matching guided pose estimation methods, challenging conditions, such as illumination changes and viewpoint changes, as well as inaccurate keypoint localization, continue to affect the performance of camera pose estimation. In this paper, we propose a novel relative camera pose regression framework that uses global features with rotation consistency and local features with rotation invariance. First, we apply a multi-level deformable network to detect and describe local features, which can learn appearances and gradient information sensitive to rotation variants. Second, we process the detection and description processes using the results from pixel correspondences of the input image pairs. Finally, we propose a novel loss that combines relative regression loss and absolute regression loss, incorporating global features with geometric constraints to optimize the pose estimation model. Our extensive experiments report satisfactory accuracy on the 7Scenes dataset with an average mean translation error of 0.18 m and a rotation error of 7.44° using image pairs as input. Ablation studies were also conducted to verify the effectiveness of the proposed method in the tasks of pose estimation and image matching using the 7Scenes and HPatches datasets.

## 1. Introduction

### 1.1. Background and Introduction

In recent years, the development of deep learning and computer vision technologies [1,2,3] has led to widespread research on camera pose estimation in both academia and industry [4,5,6]. Accurate and robust camera pose estimation is crucial for downstream tasks, such as object localization, size estimation, camera movement justification, activity recognition, and more, which can enable the development of smart living spaces. Examples of such applications include fire detection, locating ingredients for cooking robots, and planning routes to kitchens and offices. Estimating the camera’s 6 degrees of freedom (6-DoF) pose from images captured by the camera can be achieved through end-to-end deep learning [7] or feature matching from structure-based approaches [8]. By integrating advanced deep learning technology with color and depth cameras as input sensors, multi-sensor systems can assist in intelligent living.

Current image-based camera pose estimation methods are greatly affected by challenging scenes, especially illumination changes, viewpoint changes, etc. These problems lead to inaccurate image-based pose estimation. End-to-end methods based on features and descriptors, such as LIFT [9], L2-Net [10], etc., aim to improve performance by changing the network structure and order of detectors and descriptors. Regression-based methods for camera pose estimation can either learn the mapping from image pixels to absolute poses [11] or learn the relative poses of a pair of images, as in MapNet [12], PVL [13], and other methods. These methods optimize the parameters of neural networks in an end-to-end manner, enabling the use of learnable physical geometric knowledge from image pairs to regress both absolute and relative camera poses. Geometric constraints between the relative and absolute poses of the image pair are added to learn the absolute loss of a single image and the relative loss of the image pair [12]. However, models based on these methods suffer from low matching efficiency, lack robustness to challenging scenarios, and struggle with accurate keypoint extraction.

To address the aforementioned issues and leverage the geometric constraints between global feature image pairs, the model design is based on a combination of the deformable network method [14,15] (which enhances the supervision of spatial sampling locations in CNNs) and the D2-Net network. The deformable network predicts dense spatial transformations, while D2-Net simultaneously learns detectors and descriptors of key points. The novelty of the research lies in the integration of epipolar geometry, multi-level deformable convolution, a novel loss function in an end-to-end framework, and automatic hyperparameter fine-tuning during training. This paper aims to fill the gaps in the existing literature by proposing a more effective and accurate approach for camera pose estimation, which has potential applications in various downstream tasks, such as object localization, object size estimation, camera movement justification, activity recognition, and intelligent living in smart spaces. This paper mainly combines the multi-view features of local features and global features, and through strong supervision, the algorithm learns the shape changes of the image to the input local features, such as point-line structure, gradient values in different directions and dimensions, etc. Subsequently, the relevant global features are extracted and utilized for regression-based positioning. As the deformable network can calculate images of different scales simultaneously on a multi-layer convolutional network, we introduce a differential deformable network as the front-end network for feature extraction, which can combine local features that are sensitive to rotation. The global rotation consistency of invariance and global features enhances the positioning performance of the algorithm. As a result, the algorithm is robust to both static and dynamic objects.

The main contributions of the paper are as follows:

- We propose a novel end-to-end camera pose estimation framework that uses image pairs as input and leverages epipolar geometry to generate image pixel pairs for estimating the camera pose. The framework also includes the automatic fine-tuning of hyperparameters during the training process, resulting in improved accuracy and adaptability.
- We adopt a multi-level deformable convolution approach that simultaneously detects and describes the network to extract local features. This addresses the issue of sensitivity to shape information (such as scale, orientation, etc.) and inaccurate keypoint positioning, leading to more robust and accurate camera pose estimation;
- We propose a novel loss that integrates the detection and description loss based on local features with the relative pose loss function based on global features. This novel loss function enhances the accuracy of camera pose estimation by jointly optimizing local and global feature representations, leading to improved performance compared to existing methods;
- The proposed method is evaluated on benchmark datasets, including HPatches and 7Scenes. The HPatches dataset provides diverse image patches for illumination, viewpoint, and scale evaluation, while the 7Scenes dataset offers realistic indoor sequences for accuracy and stability testing. The experimental results verify the effectiveness of the proposed method for image-matching tasks and camera pose estimation tasks, and demonstrate its superiority compared to state-of-the-art methods.

### 1.2. Organization

The remainder of this paper is organized as follows. Section 2 presents a review of the related works, including localization with sparse local feature matching and camera localization with global feature regression, which provides the context for the proposed approach; Section 3 provides an overview of the dataset preprocessing steps, such as updating the depth image using the position grid, associating pixels with the color image and depth image, and introducing epipolar geometry; Section 4 describes the proposed method of the multi-level deformable network and local feature extraction based on pixel matching for camera pose estimation; Section 5 presents the experiments and discussions on the settings, multi-step image pixel reprojection, image-matching experiment on the HPatches dataset, and pose estimation experiment on the 7Scenes dataset. Finally, Section 6 summarizes the findings and potential implications of the regression-based camera pose estimation approach using multi-level local and global features.

## 2. Related Work

### 2.1. Localization with Sparse Local Feature Matching

According to the processing order of the descriptor and detector in the feature matching method, sparse local feature matching consists of the following branches: (1) detect-then-describe approaches that include keypoint detection stages with robust and efficient handcrafted detectors (e.g., SIFT (scale-invariant feature transform) [16], SUSAN (smallest univalue segment assimilating nucleus) [17]), or CNN-based invariants (Convolutional Neural Network) [18,19,20,21,22,23,24,25,26], followed by descriptor extraction on a sparse set of the detected keypoints with the help of image patch [27], Siamese CNN network [28], L2-distance [10], or second-order similarity regularization [29]. (2) The detect-and-describe approaches take an end-to-end approach to jointly learn keypoint locations and descriptors. LIFT (learned invariant feature transform) [9] uses a full-featured point-handling pipeline, including feature detecting, orientation estimating, and feature describing. LF-Net (local feature network) [30] proposes to confine a two-branch network into one branch for feature extraction in an end-to-end manner. SuperPoint [26] jointly learns keypoint detection and description, while R2D2 (repeatable and reliable detector and descriptor) [31] trains predictors of the local descriptor discriminator. ASLFeat [32] is based on D2-Net [33] and improves the perception ability of geometric invariance. DH3D [34] uses an embedding of detection and description modules in a Siamese network. (3) The describe-to-detect methods extract descriptors first and then detect keypoints. D2-Net [33] detects keypoints on a dense feature map for more stable detectors, while DELF [35] is proposed for training keypoints in a local maxima way. The above methods are computationally intensive due to the multi-stage processing periods, which rely heavily on parameter assumptions and prior knowledge. Our approach integrates the image-matching process, detection and description process, and global feature extraction process. The proposed framework can easily extract sparse local features in an end-to-end manner.

### 2.2. Camera Localization with Global Feature Regression

The regressed global features are used to compute the absolute camera pose through single monocular images or image sequences. PoseNet [11] initially regresses the 6-DoF pose through a single image. According to the loss function type, global feature-based regression methods include: (1) fixed Euclidean loss-based methods, which introduce the scaling factor for balancing the position item and orientation item [11], or add Bernoulli distributions to describe the uncertainty of localization [36]. Furthermore, LSTM [37,38] adds four LSTM units and SVS [39] adds a classification module to improve performance. (2) Learnable pose loss-based methods learn the weight pose to make the results more stable [40]. Later, the adversarial network [41] and novel DNN [13] are added to share the same loss function. (3) Relative sequence loss-based methods learn the loss from a pair of images with a geometric constraint [12]. These methods combine the absolute pose loss and the relative pose loss from an image pair, and the two terms are added with a weighting factor. Later, DGRNet [42] adopted a similar approach to MapNet [12] by extracting features from image pairs. The aforementioned methods lack accuracy in the task of pose estimation with image pairs as input and multiple parameters to optimize. Our proposed method leverages local features from image correspondences and demonstrates robustness in changing environments.

## 3. Dataset Preprocessing and Epipolar Geometry

The proposed methodology aims to estimate the camera pose by utilizing the correspondences obtained from RGB and depth image pairs as input. Data processing strategies, such as random cropping and normalization operations, are consistently applied to the fixed-step input images. Epipolar geometry [43] is employed to calculate the pixel correspondences.

### 3.1. Update Depth Image Using Position Grid

The correspondences between color image pairs are determined based on the pixel positions and intensities of the corresponding depth image pairs. We designed a position grid to assist in the processing of corner pixel identification, depth information judgment, and interpolation.

Given the width and height of the first image in the depth image pairs, we create a corresponding position grid for further computation. Specifically, for a depth image size of h×w, we construct a vector of size (2,h×w) to represent the coordinates of the position grid. The vector contains two matrices of size (h,w) each, representing the horizontal and vertical coordinates respectively. The first matrix is formed by stacking column vectors of dimension (h,1) with elements [0,h−1] in the column space *w* times, while the second matrix is formed by stacking row vectors of dimension (1,w) with elements [0,w−1] in the row space *h* times.

**Figure 1:**
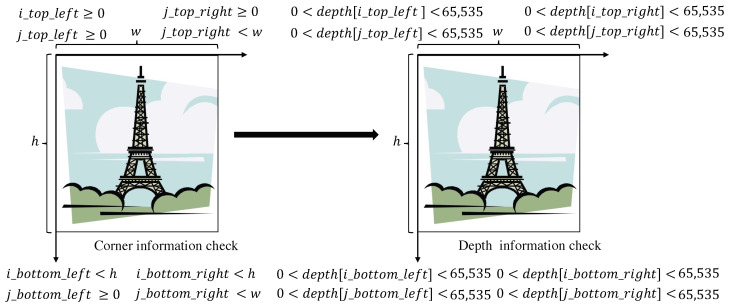
Corner information and depth information pixel judgment of the depth map.

To eliminate the coordinate positions with unqualified pixels and perform further pixel matching, we process the corner and depth value of the first depth image in the image pair. Specifically, given the first depth image and its corresponding position grid, the two dimensions of the position grid are defined as the *i* and *j* index values, respectively. Firstly, we check whether the index values of the four corners of the depth image are within the range of the image’s width and height, as shown in Figure 1. Next, we check whether the depth of the pixels represented by the index values is greater than 0 (i.e., not occluded) and less than 65,535 (the maximum value for depth information storage, corresponding to a distance of 65 me), and update the index value that conforms to the corner and depth information checks in the position grid.

After obtaining the filtered depth image and its corresponding position grid, we use weight coefficients, which are determined by the upper and lower bounds of the *i* and *j* index values, to compute new depth information values by a weighted sum of the four nearest depth values; we use bilinear interpolation to update the pixel values of the depth image. In addition, the 2D coordinates and 1D index values of the filtered depth images are stored for further conversion.

### 3.2. Associate Pixels of the Color Image and Depth Image

To obtain pixel matches between color images, we apply epipolar geometry to the depth map, camera intrinsics, and camera extrinsic parameters of the 7Scenes dataset. Epipolar geometry calculates the relationships between the 3D points and points on the projected 2D images from cameras taken from different views.

As the camera intrinsic parameters of the 7Scenes dataset were not calibrated, we followed the official instructions and set the focal length to 585, the coordinate axis tilt parameter to 0, and the principal point coordinates to (320, 240). KinectFusion provides the camera’s extrinsic parameters in the 7Scenes dataset.

**Figure 2:**
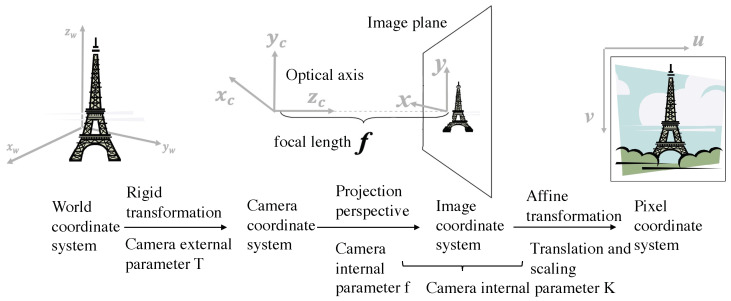
Coordinate transformation between the world coordinate system, camera coordinate system, image coordinate system, and pixel coordinate system.

**Figure 3:**
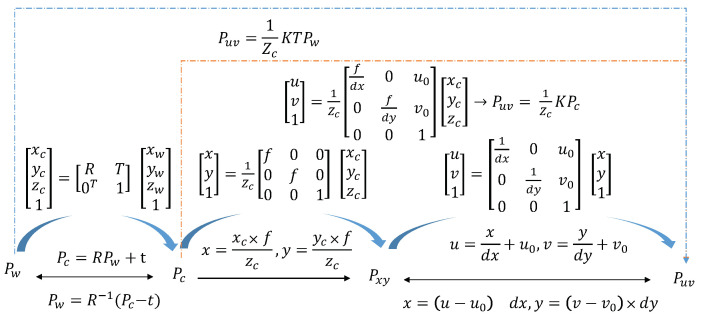
Coordinate conversion formula between different coordinate systems.

The pinhole camera model projects objects from the world coordinate system to the 2D pixel plane through the camera plane. In this model, $P_{w}={\left[x_{w},y_{w},z_{w}\right]}^{T}$, $P_{c}={\left[x_{c},y_{c},z_{c}\right]}^{T}$, $P_{xy}={\left[x,y\right]}^{T}$, and $P_{uv}={\left[u,v\right]}^{T}$ represent the same object in the world coordinate system, camera coordinate system, image coordinate system, and pixel coordinate system, respectively. The depth information is lost from the camera’s coordinate system to the image coordinate system during the projection process. Through a rigid transformation, perspective transformation, and affine transformation, the coordinate transformation can be performed in different coordinate systems. The specific transformation method and equation are shown in Figure 2 and Figure 3.

Since the same point in the real world has the same coordinates in the world coordinate system, it is possible to obtain the world coordinates from the pixel coordinates in the first image through coordinate transformation. Then, the pixel coordinates of the same point in the second image can be obtained from its world coordinates. This allows us to obtain pixel correspondences between the image pairs.

**Figure 4:**
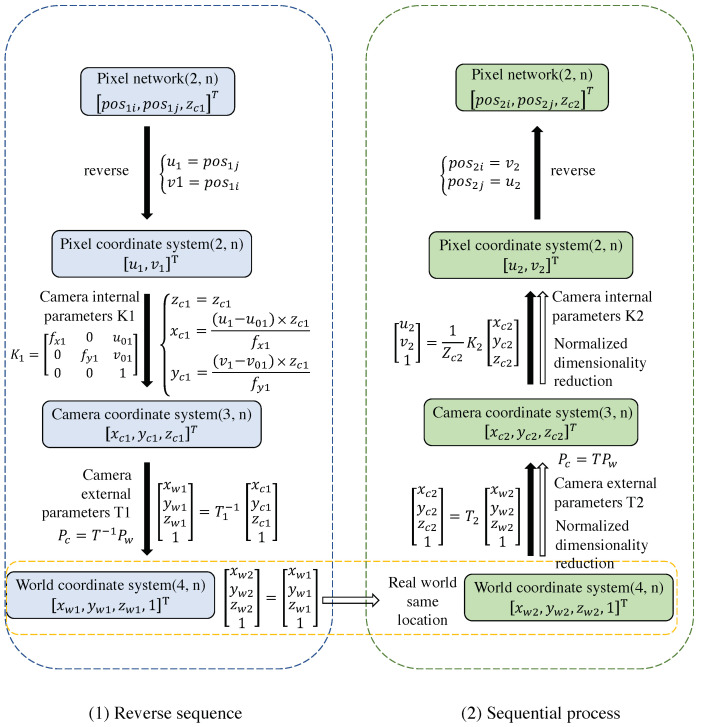
The coordinate point pair corresponds to the image pair generated by the coordinate system conversion relationship. (Here the subgraph (1) shows the reserve computation sequence of the coordinates while (2) shows the sequential process.

Figure 4 depicts the process of obtaining pixel matching between image pairs with qualified depth information. Among them, since the pixel grid coordinate system and the pixel coordinate system are orthogonal, the mutual conversation needs to exchange the positions of the two coordinate axes. Pixels of the second depth image (whose depth value difference between the transformed pixels and original pixels is greater than 0.05) are considered occluded and are filtered out. After these procedures, the filtered pixel correspondences between the color image pairs can be obtained.

## 4. Method

**Figure 5:**
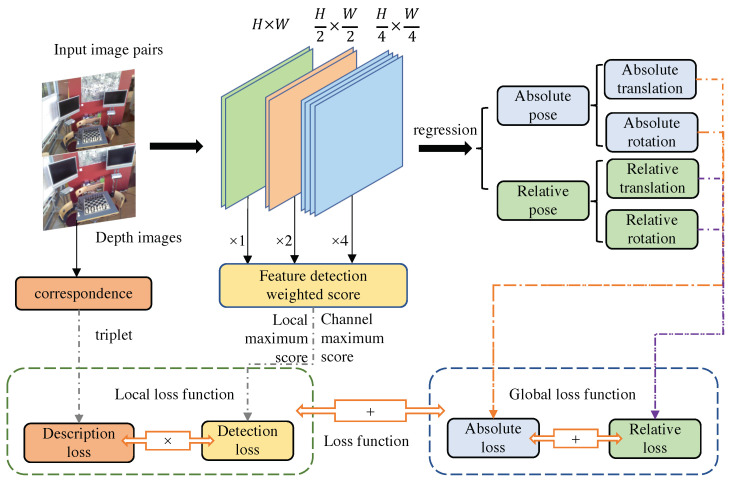
The network architecture fusing local and global features.

**Figure 6:**
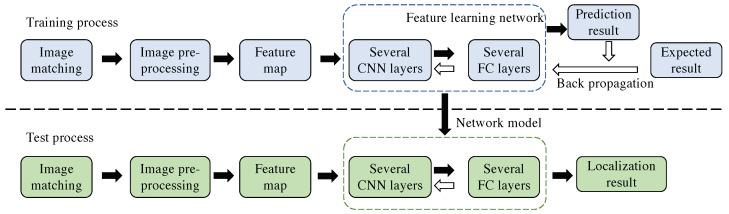
Algorithm training and testing process fusing local and global features.

This section presents the framework of the proposed method, which is an end-to-end camera pose estimation network based on relative pixel correspondences and the multi-level deformable network. We also introduce our designed loss function, which includes the global features and local description-detection features loss. Figure 5 illustrates the architecture of the network, which combines the supervision of local and global features. The input to the network is an image pair that includes related depth images and pose ground truth. The multi-level deformable network based on L2-Net is used as the feature extractor, and different image resolutions are applied in multi-convolutional layers. The feature detection score map is obtained by sampling and weighting different feature maps. The extracted features are used to regress the absolute pose and relative pose through a fully connected layer. The whole process includes four stages: data preprocessing, image feature extraction, image feature fusion, and image pose regression. The algorithm is illustrated in Figure 6 with the training and testing periods.

### 4.1. Multi-Level Deformable Network

To enhance the modeling ability of convolutional neural networks with fixed geometric structures, a deformable convolution is introduced. It learns offset locations of spatial samples in target tasks [14,15] through back-propagation and training the network in an end-to-end manner. This allows for the estimation of pixel-level local feature transformations and global shape modeling using stacked convolutional networks.

#### 4.1.1. Deformable Convolutional Network

**Figure 7:**
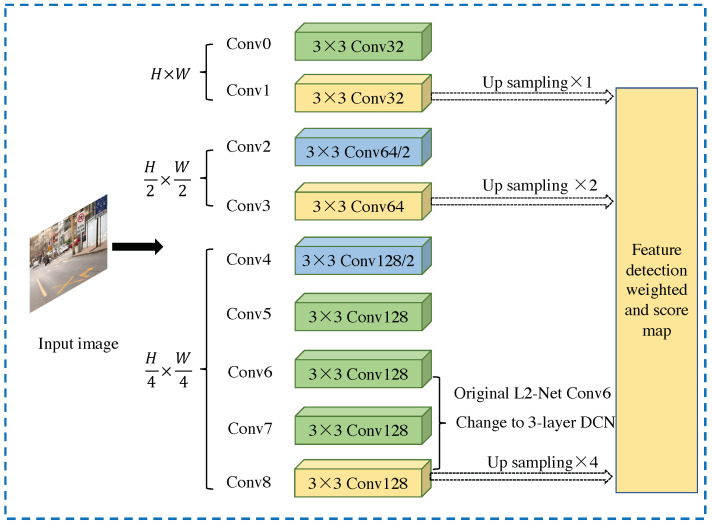
Multi-level deformable network infrastructure.

The deformable network has the ability to densely estimate local changes in the images and model the transformation of CNNs by learning the offsets added in the spatial sampling locations. The framework of the deformable network is shown in Figure 7, and it can be trained directly from scratch. To reduce the amount of calculation, the network uses the lightweight L2-Net [10] as the backbone network while changing the last 8×8 convolution layer into three 3×3 convolution layers. The network outputs a 128-dimensional feature map, which is 1/4 of the input resolution.

The goal of the deformable convolutional network (DCN) [14] is to improve the ability to model geometric changes by dynamically learning the changing receptive field. To achieve this, we use a regular grid *R* to sample the input feature map *x* in a dense and local manner [14]. The location enumeration $p_{k}$ represents a specific location on *R*. The output of a single location, $p_{0}$, on the feature map *y* can be computed as follows:

(1) $$\begin{array}{c} y\left(p_{0}\right)=\sum_{k=1}^{K}w_{k}\times x\left(p_{0}+p_{k}\right) \end{array}$$

DCN enhances the regular convolution by additionally learning the sampling offset [14] $\left\{\Delta p_{k}|k=1,\ldots ,K\right\}$, where K=|R|; the Equation (1) can be rewritten as:

(2) $$\begin{array}{c} y\left(p_{0}\right)=\sum_{k=1}^{K}w\left(p_{k}\right)\times x\left(p_{0}+p_{k}+\Delta p_{k}\right)\times \Delta m_{k} \end{array}$$

$\Delta p_{k}$ and $\Delta m_{k}$ represent the learnable offset and module scale factor of the k−th position. The range of $\Delta m_{k}$ is in [0,1], and $\Delta p_{k}$ has no constraints on the range. The bilinear interpolation could be applied to the computation of $x(p_{0}+p_{k}+\Delta p_{k})$. In the training period, the initial values of $\Delta p_{k}$ and $\Delta m_{k}$ are given 0 and 0.5, respectively [15].

#### 4.1.2. Multi-Level Feature Detection Network

Obtaining features from low-resolution feature maps may limit positioning accuracy. Restoring spatial resolution has proven effective in improving positioning accuracy, such as using other feature decoders (e.g., SuperPoint [26]) or employing dilation convolution (e.g., R2D2 [31]). However, these methods increase the number of learning hyperparameters and require significant GPU storage and computational resources. This method uses the multi-level detection method proposed by ASLFeat [32]. This method achieves the restoration of image spatial resolution in a simple and effective way by combining the multi-level feature detection using the inherent pyramid feature of the convolutional network.

Specifically, the method utilizes a feature hierarchical structure composed of several levels of $\left\{t^{\left(1\right)},t^{\left(2\right)},\ldots ,t^{\left(p\right)}\right\}$ where $\left\{1,2,\ldots ,2^{\left(p-1\right)}\right\}$ is the step size, and the detection network is applied at each level to obtain a set of detection scores $\left\{q^{\left(1\right)},q^{\left(2\right)},\ldots ,q^{\left(p\right)}\right\}$; each score map is up-sampled to have the same spatial resolution as the input image, and then combined using a weighting value:

(3) $$\begin{array}{c} \hat{s}=\frac{\sum_{p}w_{p}q^{\left(l\right)}}{\sum_{p}w_{p}} \end{array}$$

The advantages of multi-level detection are embodied in three aspects. First, it uses a multi-level detection method, which conforms to the classic space theory [44] because it has different sizes of receptive fields to locate key points; second, compared with U-Net [45], it recovers the spatial resolution without additional learning weights to achieve pixel-by-pixel accuracy. Finally, it keeps the low-level features unchanged but integrates multi-level semantic detection [46] to help preserve low-level structures, such as corners or edges. The architecture of the entire network is shown in Table 1, where the initial resolution of the input image is 256 × 256.

After performing feature extraction through the aforementioned multi-level deformable network, the subsequent multi-layer perceptron outputs the estimated posture location and rotation of the 3D feature through the fully connected layer. Since the network operates on input image pairs, a group of identical networks is copied to form a set of parallel networks that accept input image pairs. Finally, the network output contains a set of image pairs. The pose, feature map, and score feature map of the image are used in the subsequent split calculation process. Among them, the output of the last convolutional layer in the multi-level network is a feature map, and the weighted sum of the score map is transformed into a score feature map.

The specific calculation process is as follows: first, obtain the feature maps of the network conv1, conv3, and conv8 layers as input. Then, normalize the feature map by dividing each value by the largest value in the feature map. Next, fill the feature map with mirroring and perform two-dimensional average pooling with a step size of 1 and a pooling area size of 5 to obtain a feature map with the same size as the input. Subtract the normalized feature map and the pooled feature map from the average value of the pooled feature map to obtain the maximum scores on the channel and local levels, respectively. The maximum score multiplied by the maximum value is bilinearly interpolated to the original input image size to obtain the score feature map corresponding to the feature map; the weight coefficient is multiplied and the final score feature map is obtained.

The last three layers of L2-Net, conv6, conv7, and conv8, are replaced by DCN. To calculate multi-level features, conv1, conv3, and conv8 are selected. The weighted proportion in Equation (1) is $w_{i}=1,2,3$, and the expansion rate of searching for neighboring pixels is set to N(i,j)=3,2,1, respectively. The basic network of this method uses a multi-level deformable network as the feature extraction network, which will be introduced separately below.

### 4.2. Local Feature Extraction Based on Pixel Matching

In contrast to the traditional “detect first and then describe” approach, which consists of two separate stages, D2-Net [33] proposes a method that computes dense features of an image by simultaneously obtaining detector and descriptor representations. On the other hand, ASLFeat [32] has improved the measurement method by calculating the loss of local detection and description features. On this basis, this section proposes the loss of fusing the local features and global features. During the global image training process, the loss of the position and direction in positioning is returned. By weighing and calculating the global loss and local loss to minimize it, the positioning performance can be improved, satisfying both local rotation invariance and global rotation consistency. This section will introduce the process and method of local feature extraction based on pixel matching.

The loss function module includes the global feature loss and the local feature loss. The global feature loss is the weighted sum of the absolute pose loss of the query image and the relative pose loss between image pairs. The local feature loss is the combination of the descriptor loss and the detector loss. The combination is obtained by maximizing and normalizing the product by matching the corresponding positive and negative sample triple loss and the product of the local maximum score obtained in the feature map and the channel maximum score.

#### 4.2.1. Loss of Feature Descriptor

After the input training image *I* passes through the multi-level deformable convolutional network *F*, a three-dimensional tensor $F=F\left(I\right),F\in R^{h\times w\times n}$ can be obtained, where h×w is the feature map size and *n* is the number of channels. The most direct representation of the three-dimensional tensor *F* is to set the descriptor vector *d* as a dense set where $d_{ij}=F_{ij:},d\in R^{n}$. Here, i=1,…,h and j=1,…,w. Through the descriptor vector, it is easier to compare the difference between images and establish corresponding relationships using the Euclidean distance. These descriptors will be dynamically adjusted during the training phase. Even if the image contains strong appearance changes, the same set of points in the scene can produce similar descriptors. Before comparing the descriptors, it is necessary to apply L2 normalization to the descriptors: ${\hat{d}}_{ij}=\frac{d_{ij}}{\left|\right|d_{ij}{\left|\right|}_{2}}$.

**Figure 8:**
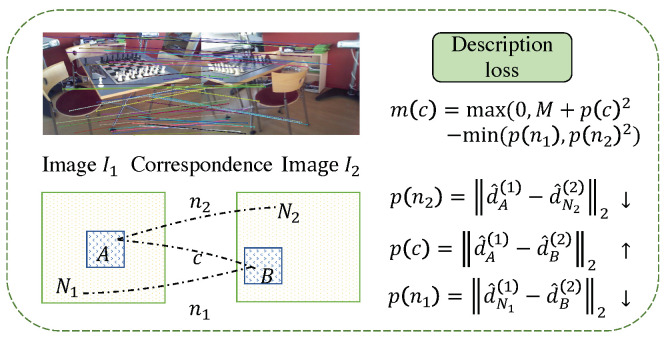
The calculation demonstration of feature description loss. (The target of the loss function is to minize $p(n_{2})$ and $p(n_{1})$, and maximize p(c)).

First, we introduce the calculation method of the ternary boundary ranking loss. Given a set of image pairs $\left(I_{1},I_{2}\right)$ and its corresponding relationship c:A←→B, where $A\in I_{1}$, $B\in I_{2}$, this loss corresponds to the distance between the pixel descriptors $\hat{d_{N1}^{\left(1\right)}}$ and ${\hat{d}}_{N2}^{\left(2\right)}$, $p\left(c\right)=\left|\right|{\hat{d}}_{A}^{\left(1\right)}-{\hat{d}}_{B}^{\left(2\right)}{\left|\right|}_{2}$ is minimized, the distance between it and the descriptor $\hat{d_{N1}^{\left(1\right)}}$, and ${\hat{d}}_{N2}^{\left(2\right)}$ of the negative sample pixel in another image is $n\left(c\right)=min(||{\hat{d}}_{A}^{\left(1\right)}-{\hat{d}}_{N2}^{\left(2\right)}|{|}_{2},||{\hat{d}}_{N1}^{\left(1\right)}-{\hat{d}}_{B}^{\left(2\right)}|{|}_{2})$. The negative sample points on the two images are defined as $N_{1}=argmin_{P\in I_{1}}\left|\right|{\hat{d}}_{P}^{\left(1\right)}-{\hat{d}}_{B}^{\left(2\right)}{\left|\right|}_{2}$$s.t.||P-{A||}_{\infty }>K$, $N_{2}=argmin_{P\in I_{2}}\left|\right|{\hat{d}}_{A}^{\left(1\right)}-{\hat{d}}_{P}^{\left(2\right)}{\left|\right|}_{2}$$s.t.||P-{B||}_{\infty }>K$. The calculation formula of the ternary boundary ranking loss is $m\left(c\right)=max(0,M+p{\left(c\right)}^{2}-n{\left(c\right)}^{2})$. The calculation diagram describing the loss is shown in Figure 8.

#### 4.2.2. Feature Detection Sub-Loss

The three-dimensional tensor F can be represented by another set of two-dimensional responses *D* [47], $D^{k}=F_{::k},D^{k}\in R^{h\times w}$, where k=1,…,n, in this interpretation, the feature extraction function *F* can be regarded as *n* different feature detection functions $D^{k}$, each of which generates a two-dimensional response graph $D^{k}$. These detection response maps are similar to the Gaussian difference (DoG) response maps obtained in the scale-invariant feature transformation (SIFT [9]), or the score maps obtained in the Harris corner detection algorithm [48].

Traditional feature detection methods (such as DoG) make the detection map sparse by suppressing the non-maximum value of the space part. Selecting the detected point (i,j) from multiple detection images $D^{k}\left(k=1,\ldots ,n\right)$ requires meeting the following criteria: in $D^{k},D_{ij}^{k}$ is the local maximum, and the value of *k* is such that $D_{ij}^{t}$ is the maximum value of t. It can be intuitively understood that for each pixel (i,j), we first select the best detector $D^{k}$ in the different channels, and then verify whether the response graph $D^{k}$ of the detector is on (i,j). There is a local maximum. Because backpropagation is required during network training, a series of scores are used to represent the detection information of pixels. First, the local maximum score is defined as the keypoint peak detection:

(4) $$\begin{array}{c} \alpha_{ij}^{k}=softplus\left(D_{ij}^{k}-\frac{\sum_{\left(i^{\prime },j^{\prime }\right)\in N\left(i,j\right)}exp\left(D_{i^{\prime }j^{\prime }}^{k}\right)}{\left|N(i,j)\right|}\right) \end{array}$$

Among them, N(i,j) is a collection of nine pixels, including the pixel (i,j) and its surroundings. The channel selection is defined as the non-maximum suppression of each descriptor on the channel:

(5) $$\begin{array}{c} \beta_{ij}^{k}=softplus\left(D_{ij}^{k}-\frac{\sum_{t}D_{ij}^{t}}{K}\right) \end{array}$$

In order to consider both the on-channel and local scores, all feature maps are multiplied and maximized to obtain a score map:

(6) $$\begin{array}{c} y_{ij}=max_{t}\alpha_{ij}^{k}\beta_{ij}^{k} \end{array}$$

The score is obtained by performing image-level normalization on the pixel point (i,j):

(7) $$\begin{array}{c} s_{ij}=\frac{y_{ij}}{\sum_{i^{\prime }j^{\prime }}y_{i^{\prime }j^{\prime }}} \end{array}$$

**Figure 9:**
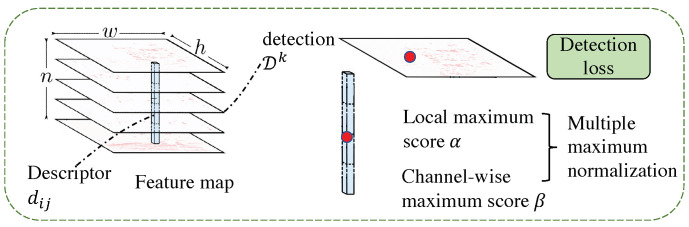
The calculation demonstration of the feature detection loss.

The schematic of the detection loss is shown in Figure 9. To make the neural network more robust to scale changes and viewpoint changes, an image pyramid is used to send the input image to the neural network at three resolutions of 0.5, 1, and 2 times, respectively. For each resolution ρ, the feature map $F^{\rho }$ is calculated. Then, the feature map of the smaller-resolution image is transferred to the feature of the larger-resolution image. The summation between feature maps of different resolutions needs to use bilinear interpolation to adjust the resolution of the feature maps to the same.

(8) $$\begin{array}{c} {\tilde{F}}^{\rho }=F^{\rho }+\sum_{y<\rho }F^{y} \end{array}$$

In order to use a single neural network to train the detection and description process at the same time, it is necessary to use a loss function that optimizes the detection and description while targeting local features, so that the key points in the detection process are repeatable in viewpoint changes and illumination changes. During the description process, each descriptor is intentionally made different from each other to avoid mismatches. The ternary boundary ranking loss is used to optimize the descriptors while maintaining their distinctiveness. To increase the optimization of the repeatability of the detector, the loss of the detection item $s_{ij}$ is added to the ternary boundary ranking loss. The detection and description processes can be optimized at the same time, so the loss function of the local feature is:

(9) $$\begin{array}{c} loss\left(I_{total}\right)=\frac{1}{K}\sum_{k\in K}\frac{s_{k}^{i}s_{k}^{j}}{\sum_{q\in K}s_{q}^{i}s_{q}^{j}}m\left(p\left(k\right),n\left(k\right)\right) \end{array}$$

#### 4.2.3. Loss Function Based on the Image Sequence for Global Features

For global features, in addition to the loss function of learnable weights that can constrain geometric information, MapNet [12] proposes the use of time constraints on image pairs. This helps to force the network to learn global features that achieve overall positioning accuracy. The method in this section uses geometric constraints and time constraints as the loss functions of the global feature, expressed as:

(10) $$\begin{array}{c} loss\left(I_{global}\right)=loss\left(I_{i}\right)+\alpha \sum_{i\neq j}loss\left(I_{ij}\right) \end{array}$$

Among them, *i* and *j* represent the index values of a pair of image pairs, $I_{ij}=\left(p_{i}-p_{j},q_{i}-q_{j}\right)$ represents the relative pose between the images $I_{j}$ and $I_{j}$, and α is the absolute pose loss obtained from a single image. The weighting factor between the relative pose loss obtained from the image pair $loss(I_{i})$ is used to describe the distance between the predicted value of the camera pose and the pose ground truth, which is defined as:

(11) $$\begin{array}{c} loss\left(I_{i}\right)=||p-p^{*}{\left|\right|}_{1}e^{-\beta }+\beta +||q-q^{*}{\left|\right|}_{1}e^{-y}+y \end{array}$$

## 5. Experiment and Discussion

### 5.1. Experimental Settings

#### 5.1.1. Datasets

The 7Scenes dataset [49] is released by Microsoft; it uses Kinect to collect indoor datasets with color maps, depth maps, and pose ground truth in 7 scenes. It is popular as a benchmark in indoor camera pose estimation experiments.

The HPatches dataset [50] includes 116 image sequences and the ground truth of homography matrices, which could be used to evaluate the extraction performance of local descriptors. The 57 sequences include illumination conversion and the 59 sequences include viewpoint/occlusion conversion.

#### 5.1.2. Implementation Details

The experiment was implemented with PyTorch [51] on NVIDIA Titan X GPU [52]. The following experiment parameters were chosen based on empirical experimentation:

- Batch size of 4. This choice balances computational efficiency and memory usage;
- Number of matching correspondences of 128. This value is commonly used in related literature for keypoint matching tasks [33];
- Training iterations of 1000. This value was determined based on empirical experimentation to achieve optimal convergence and performance;
- Balancing factors between detection loss and description loss, absolute loss and relative loss, and local loss and global loss, all set to 1. These values were chosen to give equal importance to different components of the loss function, which could also achieve better performance according to the experiments;
- Initial learning rate of $1\times 10^{-5}$ for the first 100 iterations, divided by 5 for every 100 iterations thereafter. This learning rate scheduling was determined based on empirical experimentation to achieve optimal training progress and convergence.

We use a batch size of 4, with 128 matching correspondences. The balancing factors between the detection loss and description loss, the absolute loss and the relative loss, and the local loss and global loss are all set to 1. The training iterations are set to 1000, with an initial learning rate of $1\times 10^{-5}$ for the first 100 iterations, and then divided by 5 for every 100 iterations. The backbone network is trained from scratch without pretraining on the classification dataset. The input images are uniformly scaled to 256 pixels on the short side and then randomly cropped to 256×256. For every iteration, a pair of images with a frame index difference of 10 is selected. The stochastic gradient descent optimizer is used with the Adam [53] solver for fine-tuning. During inference, the input images are scaled to 256 pixels on the short side and then center-cropped to 256×256.

### 5.2. Multi-Step Image Pixel Reprojection

Given the index gap of 10 and a total of 2 images, we can obtain a set of image pairs from the 7Scenes dataset, which includes depth images, color images, and camera intrinsic and extrinsic parameters. Through image processing and epipolar geometry, the pixel correspondences of the image pairs could be computed.

**Figure 10:**
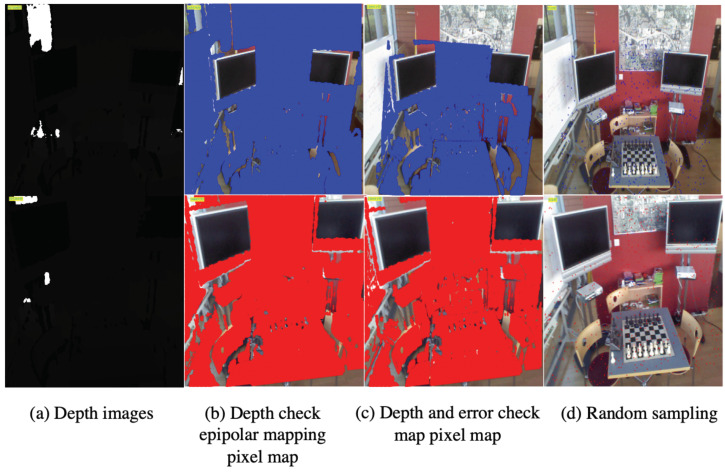
The calculation process of the corresponding relationship between image pairs.

**Figure 11:**
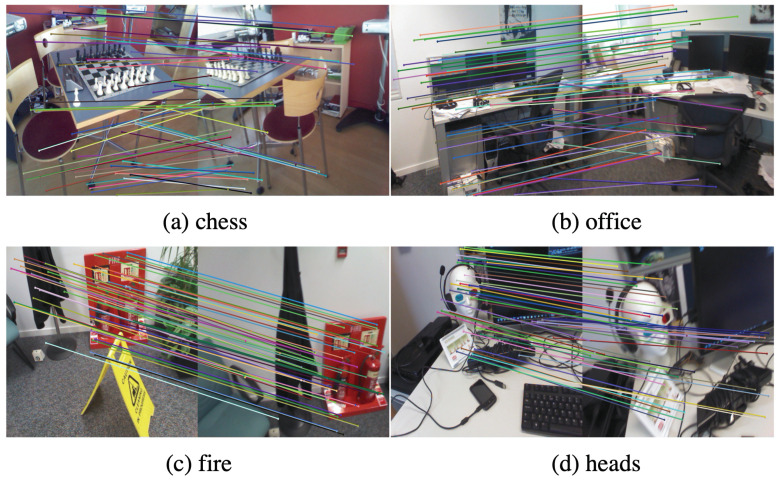
Schematic diagram of matching relationships in different scenes.

Taking the image pair with an index gap of 400 as an example, the process is illustrated in Figure 10. The initial number of pixels for each image in the pair is h×w=320×640 = 307,200. Firstly, we filter out invalid pixels using the depth check of the first depth image resulting in 245,574 pixels. Secondly, we obtain the corresponding pixel position in the second depth image using epipolar geometry, resulting in 245,574 pixels. Then, we filter out pixels with invalid depth values and corner indices resulting in 148,857 pixels. We further filter out pixels with a projected depth difference greater than 0.05 m compared to their own depth resulting in 126,411 pixel pairs. Finally, we randomly sample 512 pixel pairs. The matching correspondences are shown in Figure 11.

### 5.3. Image-Matching Experiment on HPatches Dataset

We evaluate the performance of local descriptors on the HPatches dataset using the following metrics: (1) keypoint repeatability (%Rep.): the ratio of potential matches in the co-visible view; (2) descriptor matching score (%MS): the ratio of correct matches and the minimum number of keypoints in the co-visible view; (3) mean average accuracy (%MMA): the ratio of correct matches to potential matches. A matching pair is defined as the nearest neighbors after searching, and the distance between the points is less than the error threshold. For the above indicators, Table 2 compares the average values of image pairs in the dataset with SuperPoint [26] and D2-Net [33]. SuperPoint [26] is a widely recognized and commonly used method for keypoint detection and description, known for its repeatability and accuracy in challenging scenarios. D2-Net [33] is another state-of-the-art method that has demonstrated excellent performance in local feature extraction, matching, and camera pose estimation tasks.

**Figure 12:**
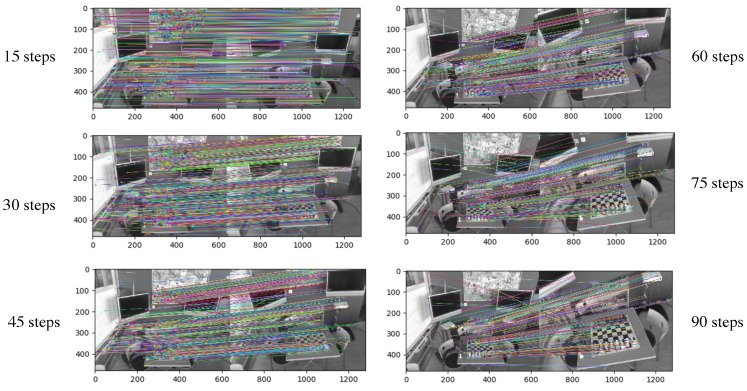
Schematic diagram illustrating the use of SIFT features for matching.

Figure 12 compares the multi-step matching keypoints results by SIFT features and the multi-step matching method in the chess scene of the 7Scenes dataset. This step represents the frame index gap. The number of keypoints from SIFT features decreases as the step increases, while the keypoints from the multi-step matching could provide constant matches within a given range, which improves the robustness of image matching and the reliability of gradient values.

It is essential to obtain robust and accurate image-matching results efficiently in challenging environmental conditions. The most popular image-matching methods could be divided into sparse matching (including detection and description processes) and dense matching (including description processes). Table 3 summarizes the process, advantages, and disadvantages of various public matching methods. Detect-then-describe methods have low robustness due to the low-dimensional features of local detectors being sensitive to pixel intensities. The dense matching methods perform well in changing illumination areas; however, the matching memory and time consumption are high.

### 5.4. Pose Estimation Experiment on 7Scenes Dataset

In order to verify the performance of our proposed network on the pose estimation task, we conducted experiments and compared the results with several competing methods that use multiple images or videos as input on the 7Scenes dataset. VidLoc [54], MapNet [12], and LSG [55] were selected for comparing the translation (in m) and rotation (in ${}^{\circ }$) errors. As shown in Table 4, our method achieves better performance with smaller pose errors compared to other related methods, which confirms the effectiveness of the proposed loss function and pixel constraints.

Furthermore, Table 5 presents a comparison of different methods that use multiple images or video as input in terms of robustness, type of graphics card, input image pixel values, processing time per image in milliseconds, and network model size. Our proposed method shows competitive performance in terms of time consumption, with smooth time and local features, and demonstrates robustness in motion blur (correspondences from image pairs could justify moving objects) and without drift (relative pose could query geometric constraints of image pairs and reduce drift). Without pre-training, the size of our network model is 60 Mb. Compared to VidLoc, the time consumption of our method for testing each image is significantly lower at 10.2 milliseconds.

To evaluate the effectiveness of each module used in the network, and to quickly conduct the experiment, we select the heads scene of the 7Scenes dataset, which has the smallest number of images, and the results from the heads scene could represent the performance of the 7Scenes dataset. The ablation study experiment was conducted for 100 iterations with a learning rate of $1\times 10^{-6}$. Since the local loss module could only be used with the output of the multi-level deformable network, we compared the pose estimation results with ResNet and multi-level deformable networks, as well as different weightings of the global loss and local loss modules. Table 6 shows that the multi-level deformable network and the combination of global loss and local loss could obtain smaller pose errors. Increasing the weight of the local loss could slightly improve the pose estimation results.

## 6. Conclusions

In this paper, we propose a regression-based camera pose estimation framework that consists of a multi-level deformable network for feature extraction and a loss function that fuses multi-view features with both local rotation invariance and global rotation consistency. To address challenges such as changing environments and motion blur in datasets, we design the feature extraction network and multi-level network to be robust and accurate. Our experiments on the 7Scenes and HPatches datasets show that our proposed network outperforms competing methods in accuracy and robustness. We demonstrate that correspondences produced by camera sensors, including RGB and depth cameras, can outperform local detection and description optimization integrated with global feature supervision, which leverages the rotation consistency of global features and the rotation invariance of local features. Moreover, the features captured within global and local supervision are also suitable for image matching. In future work, we will apply the learnable balancing factor to the loss to improve the model scalability and portability, and will try to add other common sensors, e.g., IMUs, to improve indoor localization performance and apply these methods to robot navigation and planning to enable smarter living.

## Figures and Tables

**Table 1:** Architecture parameter settings based on the L2-Net network. (Here means this layer is applied in the convolution layer.)

Layer	Input Channel	Output Channel	Kernel Size	Stride	Resolution	BN Layer	ReLU Layer	Padding	Dilation
Input	3				×1				
Conv0	3	32	3	1	×1	✔	✔	1	1
Conv1	32	32	3	1	×1	✔	✔	1	1
Conv2	32	64	3	2	×1/2	✔	✔	1	1
Conv3	64	64	3	1	×1/2	✔	✔	1	1
Conv4	64	128	3	2	×1/4	✔	✔	1	1
Conv5	128	128	3	1	×1/4	✔	✔	1	1
Conv6	128	128	3	1	×1/4	✔	✔	1	1
Conv7	128	128	3	1	×1/4	✔	✔	1	1
Conv8	128	128	3	1	×1/4			1	1

**Table 2:** Comparison of the local descriptor matching performance between this method and other methods on the HPatches dataset (HPatches dataset error threshold @4px).

	%Rep.	%M.S.	%MMA
SuperPoint [26]	45.80	31.23	39.82
D2-Net [33]	47.86	23.58	43.00
This method	72.33	42.58	68.31

**Table 3:** The process, advantages, and disadvantages of common sparse-matching methods and dense-matching methods.

	Sparse Matching		Dense Matching
	**Detect-then-Describe**	**Detect-and-Describe**	**Detect**
Process	Detect the keypoints of the image; extract the descriptors from the image patches around the keypoint; output the compact representation of the image patch	Extract descriptors and keypoints on the feature map; detect the high-dimensional keypoints with locally unique descriptors.	Perform the description stage densely on the entire image.
Advantages	High matching and storage efficiency, keypoints are sensitive to low-dimensional information, and high positioning accuracy.	Robust to challenging environments, efficient storage and matching.	Robust dense descriptors for environmental changes.
Disadvantages	Poor performance in challenging environments (weak textures, etc.), poor repeatability in keypoint detection.	Dense descriptors lead to low computational efficiency, and the accuracy of key points obtained by detectors based on high-dimensional information is not high.	High matching time consumption and memory.

**Table 4:** Localization errors of the fusion of local and global features and other methods in the 7Scenes dataset.

Methods	Chess	Fire	Heads	Office	Pumpkin	Kitchen	Stairs
PoseNet [11]	0.32 m, 8.12${}^{\circ }$	0.47 m, 14.4${}^{\circ }$	0.29 m, 12.0${}^{\circ }$	0.48 m, 7.68${}^{\circ }$	0.47 m, 8.42${}^{\circ }$	0.59 m, 8.64${}^{\circ }$	0.47 m, 13.8${}^{\circ }$
Dense PoseNet [11]	0.32 m, 6.60${}^{\circ }$	0.47 m, 14.0${}^{\circ }$	0.30 m, 12.2${}^{\circ }$	0.48 m, 7.24${}^{\circ }$	0.49 m, 8.12${}^{\circ }$	0.58 m, 8.34${}^{\circ }$	0.48 m, 13.1${}^{\circ }$
Bayesian PoseNet [36]	0.37 m, 7.24${}^{\circ }$	0.43 m, 13.7${}^{\circ }$	0.31 m, 12.0${}^{\circ }$	0.48 m, 8.04${}^{\circ }$	0.61 m, 7.08${}^{\circ }$	0.58 m, 7.54${}^{\circ }$	0.48 m, 13.1${}^{\circ }$
LSTM PoseNet [56]	0.24 m, 5.77${}^{\circ }$	0.34 m, 11.9 ${}^{\circ }$	0.21 m, 13.7${}^{\circ }$	0.30 m, 8.08${}^{\circ }$	0.33 m, 7.00${}^{\circ }$	0.37 m, 8.83${}^{\circ }$	0.40 m, 13.7 ${}^{\circ }$
Hourglass PoseNet [56]	0.15 m, 6.17${}^{\circ }$	0.27 m, 10.84${}^{\circ }$	0.19 m, 11.63${}^{\circ }$	0.21 m, 8.48${}^{\circ }$	0.25 m, 7.01${}^{\circ }$	0.27 m, 10.15${}^{\circ }$	0.29 m, 12.46${}^{\circ }$
BranchNet [57]	0.18 m, 5.17${}^{\circ }$	0.34 m, 8.99${}^{\circ }$	0.20 m, 14.15${}^{\circ }$	0.30 m, 7.05${}^{\circ }$	0.27 m, 5.10${}^{\circ }$	0.33 m, 7.40${}^{\circ }$	0.38 m, 10.26${}^{\circ }$
Geo.PoseNet [40]	0.14 m, 4.50${}^{\circ }$	0.27 m, 11.8${}^{\circ }$	0.18 m, 12.1${}^{\circ }$	0.20 m, 5.77${}^{\circ }$	0.25 m, 4.82${}^{\circ }$	0.24 m, 5.52${}^{\circ }$	0.37 m, 10.6${}^{\circ }$
AdPR [58]	0.12 m, 4.8${}^{\circ }$	0.27 m, 11.6${}^{\circ }$	0.16 m, 12.4${}^{\circ }$	0.19 m, 6.8${}^{\circ }$	0.21 m, 5.2${}^{\circ }$	0.25 m, 6.0${}^{\circ }$	0.28 m, 8.4${}^{\circ }$
APANet [41]	N/A, N/A	0.21 m, 9.72${}^{\circ }$	0.15 m, 9.35${}^{\circ }$	0.15 m, 6.69${}^{\circ }$	0.19 m, 5.87${}^{\circ }$	0.16 m, 5.13${}^{\circ }$	0.16 m, 11.77${}^{\circ }$
Geo.PoseNet (reprojection) [40]	0.13 m, 4.48${}^{\circ }$	0.27 m, 11.3${}^{\circ }$	0.17 m, 13.0${}^{\circ }$	0.19 m, 5.55${}^{\circ }$	0.26 m, 4.75${}^{\circ }$	0.23 m, 5.35${}^{\circ }$	0.35 m, 12.4${}^{\circ }$
GPoseNet [59]	0.20 m, 7.11${}^{\circ }$	0.38 m, 12.3${}^{\circ }$	0.21 m, 13.8${}^{\circ }$	0.28 m, 8.83${}^{\circ }$	0.37 m, 6.94${}^{\circ }$	0.35 m, 8.15${}^{\circ }$	0.37 m, 12.5${}^{\circ }$
MapNet [12]	0.08 m, 3.25${}^{\circ }$	0.27 m, 11.7${}^{\circ }$	0.18 m, 13.3${}^{\circ }$	0.17 m, 5.15${}^{\circ }$	0.22 m, 4.02${}^{\circ }$	0.23 m, 4.93${}^{\circ }$	0.30 m, 12.1${}^{\circ }$
LSG [55]	0.09 m, 3.28${}^{\circ }$	0.26 m, 10.92${}^{\circ }$	0.17 m, 12.70${}^{\circ }$	0.18 m, 5.45${}^{\circ }$	0.20 m, 3.69${}^{\circ }$	0.23 m, 4.92${}^{\circ }$	0.23 m, 11.3${}^{\circ }$
VidLoc [54]	0.18 m, N/A	0.26 m, N/A	0.14 m, N/A	0.26 m, N/A	0.36 m, N/A	0.31 m, N/A	0.26 m, N/A
This method	0.08 m, 3.19${}^{\circ }$	0.25 m, 10.89${}^{\circ }$	0.14 m, 12.5${}^{\circ }$	0.16 m, 5.15${}^{\circ }$	0.20 m, 4.01${}^{\circ }$	0.21 m, 4.91${}^{\circ }$	0.25 m, 11.2${}^{\circ }$

**Table 5:** Comparison of experimental qualitative results between the method of fusing local and global features and other methods.

Methods	Input	Robustness	Graphics Card	Pixel Values	Time (ms)
VidLoc [54]	Video	Temporal smooth	Titan X	256×256	18∼43
MapNet [12]	Image pair, video	Locally smooth drift-free	/	256×256	9.4
LSG [55]	Image pair	Posture uncertainty caused by content enhancement	Nvidia 1080Ti	256×256	unknown
This method	Image pair, depth image	Time smooth, motion blur, no drift	Nvidia Titan X GPU	256×256	10.2

**Table 6:** Positioning errors in the Heads scenario of the 7Scenes dataset using different networks and loss functions. (Here means that this network module and loss function(s) are used in the framework.)

Networks		Modules			7Scenes Dataset
**ResNet34**	**Multi-Level** **Deformation** **Network**	**Global** **Loss**	**Local** **Loss**	**Local** **Loss** **Weight**	**Heads Scenes**
✔		✔			0.25 m, $17.5^{\circ }$
	✔		✔		0.24 m, $16.2^{\circ }$
	✔	✔	✔	1.0	0.15 m, $12.6^{\circ }$
	✔	✔	✔	2.0	0.14 m, $12.5^{\circ }$

## Data Availability

Publicly available datasets were analysed in this study. These datasets can be found here: 7Scenes dataset: https://www.microsoft.com/en-us/research/project/rgb-d-dataset-7-scenes/, (accessed on 1 January 2013); HPatches dataset: https://github.com/hpatches/hpatches-dataset, (accessed on 19 April 2017).

## Abbreviations

The following abbreviations are used in this manuscript:

*I*	image
*h*	height of the image
*w*	width of the image
*i*	index value of the image height
*j*	index value of the image width
$P_{w}$	the point coordinates $\left(x_{w},y_{w},z_{w}\right)$ in the world coordinate system
$P_{c}$	the point coordinates $\left(x_{c},y_{c},z_{c}\right)$ in the camera coordinate system
$P_{xy}$	the point coordinates (x,y) in the image coordinate system
$P_{uv}$	the point coordinates (u,v) in the pixel coordinate system
*f*	the focal length in the pinhole model
*T*	camera external parameter matrix
*K*	camera internal parameter matrix
*x*	feature map
*R*	regular grid
$p_{k}$	the enumeration of the location in *R*
$\Delta p_{k}$	the learnable offset
$\Delta m_{k}$	the module scale factor of the k−th position
$w_{i}$	the weight factor in different convolutional layers
N(i,j)	the expansion rate of searching for neighboring pixels
*F*	the tensor obtained through the multi-level deformable convolutional network
*n*	number of channels
*d*	descriptor vector
*N*	negative sample points in the image *I*
*D*	two-dimensional response
ρ	resolution
